# 774 Digital Interventions for Social Participation in Adults with Long-term Physical Conditions: A Systematic Review

**DOI:** 10.1093/jbcr/irac012.327

**Published:** 2022-03-23

**Authors:** Huan Deng, Lauren J Shepler, Callie Abouzeid, Jason W Hamner, Hannah W Mercier, J Andrew Taylor, Lewis E Kazis, Mary D Slavin, Colleen M Ryan, Jeffrey C Schneider

**Affiliations:** Spaulding Rehabilitation Hospital, Harvard Medical School, Boston, Massachusetts; Spaulding Rehabilitation Hospital, Boston, Massachusetts; Spaulding Rehabilitation Hospital, Boston, Massachusetts; Spaulding Rehabilitation Hospital, Boston, Massachusetts; Stony Brook University, Stony Brook, New York; Spaulding Rehabilitation Hospital, Harvard Medical School, Cambridge, Massachusetts; Boston University School of Public Health, Spaulding Rehabilitation Hospital, Harvard Medical School, Boston, Massachusetts; Boston University School of Public Health, Boston, Massachusetts; Harvard Medical School, Boston, Massachusetts; Spaulding Rehabilitation Hospital, Harvard Medical School, Massachusetts General Hospital, Boston, Massachusetts

## Abstract

**Introduction:**

Burn survivors experience significant social participation challenges in their recovery. However, enrolment and compliance with face-to-face interventions for such issues are often limited by time, location, and financial resources. Digital technologies are increasingly utilized in healthcare and provide a flexible, accessible, and low-cost treatment option. Given the sparse literature on this topic in the burn field, this review evaluated digital interventions for social participation in adults with long-term physical conditions to inform future use in the burn population.

**Methods:**

MEDLINE, EMBASE, CINAHL and PsycINFO databases were searched using keywords and Medical Subject Headings (MeSH) terms related to ‘digital intervention’ and ‘social participation’ for studies published in English between January 2010 and May 2021. Studies that adopted digital technology interventions to improve social participation in adults with long-term physical conditions were included. Study quality was evaluated using Oxford Levels of Evidence. Data on study methodology, digital intervention and findings related to social participation were summarized.

**Results:**

The search yielded a total of 4646 articles, of which 158 were full-text screened and 14 met inclusion and exclusion criteria. There were five randomized controlled trials, two non-randomized clinical trials and seven one-group pretest-posttest clinical trials. Twenty-five different measurement tools were utilized to assess social participation and two of them were used twice. Three types of digital interventions were implemented to improve social participation: group support, individual skill training or counselling, and education and support. The group support intervention developed a social network among affected people through videoconference, app, or virtual reality platform (3 of 4 studies with positive results). Individual skill training or counselling utilized phone calls or videoconference to help participants with activity participation and interpersonal relationships (2 of 6 studies with positive results). The education and support intervention used messages and website information to increase participants’ knowledge and provide support (3 of 3 studies with positive results).

**Conclusions:**

This review presents evidence of different digital interventions’ effect on improving social participation in adults with long-term physical conditions. However, the existing literature is limited by the heterogeneity of outcome measures and varied methodology quality that preclude larger generalizations.

